# Site-Directed Mutagenesis of IRX9, IRX9L and IRX14 Proteins Involved in Xylan Biosynthesis: Glycosyltransferase Activity Is Not Required for IRX9 Function in Arabidopsis

**DOI:** 10.1371/journal.pone.0105014

**Published:** 2014-08-13

**Authors:** Yanfang Ren, Sara Fasmer Hansen, Berit Ebert, Jane Lau, Henrik Vibe Scheller

**Affiliations:** 1 Joint Bioenergy Institute, Lawrence Berkeley National Laboratory, Berkeley, California, United States of America; 2 College of Agriculture, Guizhou University, Guiyang, China; 3 Department of Plant and Environmental Sciences, Faculty of Science, University of Copenhagen, Frederiksberg, Denmark; 4 Department of Plant and Microbial Biology, University of California, Berkeley, California, United States of America; University of Massachusetts Amherst, United States of America

## Abstract

Xylans constitute the main non-cellulosic polysaccharide in the secondary cell walls of plants. Several genes predicted to encode glycosyltransferases are required for the synthesis of the xylan backbone even though it is a homopolymer consisting entirely of β-1,4-linked xylose residues. The putative glycosyltransferases IRX9, IRX14, and IRX10 (or the paralogs IRX9L, IRX14L, and IRX10L) are required for xylan backbone synthesis in Arabidopsis. To investigate the function of IRX9, IRX9L, and IRX14, we identified amino acid residues known to be essential for catalytic function in homologous mammalian proteins and generated modified cDNA clones encoding proteins where these residues would be mutated. The mutated gene constructs were used to transform wild-type Arabidopsis plants and the *irx9* and *irx14* mutants, which are deficient in xylan synthesis. The ability of the mutated proteins to complement the mutants was investigated by measuring growth, determining cell wall composition, and microscopic analysis of stem cross-sections of the transgenic plants. The six different mutated versions of IRX9 and IRX9-L were all able to complement the *irx9* mutant phenotype, indicating that residues known to be essential for glycosyltransferases function in homologous proteins are not essential for the biological function of IRX9/IRX9L. Two out of three mutated IRX14 complemented the *irx14* mutant, including a mutant in the predicted catalytic amino acid. A IRX14 protein mutated in the substrate-binding DxD motif did not complement the *irx14* mutant. Thus, substrate binding is important for IRX14 function but catalytic activity may not be essential for the function of the protein. The data indicate that IRX9/IRX9L have an essential structural function, most likely by interacting with the IRX10/IRX10L proteins, but do not have an essential catalytic function. Most likely IRX14 also has primarily a structural role, but it cannot be excluded that the protein has an important enzymatic activity.

## Introduction

Xylan is the most abundant non-cellulosic polysaccharide in plants, comprising around 30% of the biomass. The structure of xylan is a β-1,4-linked backbone of d-xylose residues, decorated with various substituents. Glucuronic acid residues and arabinose substitutions are the most common monosaccharide substituents. The glucuronic acid residues are often 4-*O*-methylated and acetylation of the xylose residues at C-2 or C-3 positions is also a common modification [1]. Because of the importance of xylan, many studies have addressed genes and proteins involved in the biosynthesis, but it has turned out to be unexpectedly challenging and progress has been very slow. Identification of enzymes required for adding the substitutions has been more successful, and several glucuronosyltransferases, arabinosyltransferases, xylosyltransferases and actetyltransferases have now been reported [1]–[7].

The backbone synthesis has been more difficult to elucidate. Mutations in the Arabidopsis genes *Irregular Xylem* (*IRX)7* (and the homolog *IRX7L*), *IRX8* and *PARVUS* lead to decreased xylan content, but those plants still make xylan with extended backbones and it is suggested that this set of genes might be involved in synthesizing a primer or the reducing end oligosaccharide structure found in many plants [8]. In contrast, mutations in the genes *IRX9* and *IRX14*, which are members of Glycosyltransferase family (GT)43, or *IRX10* belonging to GT47 result in decreased xylan synthase activity, reduced xylan content, and xylan with short backbones [9]–[12].

In Arabidopsis all three genes have close homologs (*IRX9L*, *IRX14L*, *IRX10L*) that are partially functionally redundant with IRX9, IRX14 and IRX10, respectively [9], [12]. The homologs IRX9/IRX9L, IRX14/IRX14L and IRX10/IRX10L appear to differ mostly in their organ specific expression patterns. Still, the proteins may not be completely redundant as e.g. overexpression lines of *IRX9L* in the *irx9* mutant background complemented the morphological phenotype but showed less xylosyltransferase activity than the wild type and varied in stem breaking strength [11]–[13]. However, it is difficult to conclude if this is due to too low expression in important target cells or to a difference in biochemical function of the two proteins. The double mutants *irx9*/*irx9L* and *irx14*/*irx14L* show an almost lethal phenotype with severe growth inhibition [12]. IRX9 and IRX14 are not redundant, as *irx9* mutants cannot be complemented by overexpression of IRX14 or IRX14L and vice versa [11], [12]. That three glycosyltransferases are required to catalyze the formation of a homopolymer consisting entirely of β-1,4-linked xylose residues is surprising. Since no in vitro activity has been reported for the individual proteins it is unclear if one protein possesses the main activity with the other proteins having an accessory or structural role, or if all proteins are active at the same time. Overexpression of IRX9 leads to increased xylan synthase activity in Arabidopsis plants [11], and other reports have shown a synergistic effect of IRX9 and IRX14 overexpression on xylan synthase activity [15]. However, these studies do not prove that IRX9 and/or IRX14 are the actual glycosyltransferases responsible for the activity as their overexpression could also stimulate other proteins. For example one may hypothesize that IRX9, IRX14 and IRX10 function together in a complex and overexpression of IRX9 alone or together with IRX14 could stabilize the complex and lead to more activity of IRX10 and IRX10L. This outcome could be expected if IRX10/IRX10L were the catalytic subunits but the amount of IRX9 and IRX14 were limiting for formation of the complex. The idea that the proteins are somehow interacting in a complex has been proposed many times, but unambiguous evidence has been hard to obtain [1], [16].

An essential anchoring rather than catalytic function of IRX9/IRX9L and IRX14/IRX14L is in agreement with a transcriptomic study of psyllium seeds (*Plantago ovata*), which are very rich in xylan [17]. Several *IRX10* homologs are expressed in the seeds, while *IRX9* and *IRX14* homologs have very low expression [18]. Interestingly, the most highly expressed *IRX10* homolog in psyllium (GenBank accession AGQ53953) encodes a protein with a strongly predicted N-terminal membrane anchor as in typical Type 2 membrane proteins. In contrast, the IRX10 and IRX10L proteins in Arabidopsis are not predicted to have any transmembrane helices, suggesting that they need to interact with other Golgi-resident proteins to be retained in the Golgi apparatus. Thus, these studies have led to the suggestion that IRX10 orthologs are the main or only catalytic proteins for xylan backbone biosynthesis, while IRX9 and IRX14 orthologs have accessory functions. Obviously, even if this is the case in psyllium seed mucilage formation, it need to be the case for other tissues in psyllium where *IRX9* and *IRX14* homologs are expressed or in other plant species.

We noted two peculiar properties of IRX9. Firstly, the *irx9-2* T-DNA mutant has a much milder phenotype than the *irx9-1* mutant. The *irx9-1* insertion is in the first intron and the mutant is likely a true knock-out. The *irx9-2* insertion is in the second exon in the first half of the protein, and if a protein is made in the mutant it would only contain the transmembrane domain and the linker region, whereas almost the entire domain predicted to contain catalytic activity would be missing. Secondly, IRX9 has an unusual amino acid sequence where the DxD motif that is found in most glycosyltransferases with a GT-A fold is not conserved. Instead in IRX9 the corresponding amino acid sequence is ‘GLN’ (Fig. S1). In contrast, in IRX9L and in IRX14/IRX14L the DxD motif is conserved (Fig. S1). These observations led us to suspect that IRX9 may not have an essential catalytic function and instead have a primary role in organizing and assembling a xylan synthase complex.

To probe the function of IRX9, IRX9L and IRX14 we mutagenized the proteins in specific amino acid residues known to be required for glycosyltransferase activity in mammalian GT43 homologs and determined if the mutated proteins could complement *irx9* and *irx14* mutants in Arabidopsis. Our results show that none of the mutations in regions that would be required for catalytic activity had any effect on the ability of IRX9 and IRX9L to complement the *irx9* mutant phenotype. In contrast, some but not all mutations in IRX14 did affect the complementation of *irx14* mutants. Thus, we conclude that IRX9 and IRX9L are not catalytically active and their main function is primarily or exclusively structural. For IRX14 our results demonstrate the importance of the DxD motif, which has been found to mainly interact with the phosphate groups of nucleotide sugar donors, but other residues presumed to be involved in catalysis are poorly conserved and seem not to be required for IRX14 function. Thus, the nucleotide sugar binding function of IRX14 is important but the protein is unlikely to have an essential catalytic role.

## Materials and Methods

### Plant Lines and Plant Growth Conditions

*Arabidopsis thaliana* accession Columbia-0 (Col-0) and T-DNA insertion lines (*irx9-2*, SALK_057033 and *irx14*, SALK_038212) were obtained from the Arabidopsis Biological Resource Center (ABRC, Ohio State University). Plants were grown on soil in a growth chamber under long-day conditions (16-h light/8-h dark cycle) at 22°C and 60% relative humidity after being stratified at 4°C for 4 days. Arabidopsis plants were transformed using *Agrobacterium tumefaciens* PGV 3805 via the floral dip method as described by Clough and Bent [19]. For BASTA selection seeds were germinated on soil as described above and 1-week-old seedlings were sprayed every 2 days for a total of five times with a glufosinate-ammonium (Crescent Chemical) solution (40 mg/mL). Resistant plants were transferred to new pots and grown as described above.

### Bioinformatics and Sequence Identification

All GT43 proteins from *Arabidopsis thaliana* (At), rice (*Oryza sativa*, Os), poplar (*Populus trichocarpa*, Pt), spikemoss (*Selaginella moellendorffii*, Sm) and human (*Homo sapiens*, Sp) were selected for multiple sequence alignment. The protein sequences have the following Genbank accession numbers (locus numbers for Arabidopsis): AtIRX9 (AT2G37090), AtIRX9L (AT1G27600), AtIRX14 (AT4G36890), AtIRX14L (AT5G67230), Os01g06450 (AHW98784), OsIRX9L/Os01g48440 (NP_001043846), Os03g17850 (ABF95369), Os04g01280 (NP_001051997), Os04g55670 (AHW98789), Os05g03174 (AHW98781), Os05g48600 (NP_001056301), OsIRX14/Os06g47340 (AHW98790), OsIRX9/Os07g49370 (AHW98783), Os10g13810 (AAP52726), Pt002G107300 (XP_002301102), Pt005G141500 (XP_002306485), Pt006G131000 (XP_006381488), Pt006G240200 (XP_002309550), Pt007G047500 (XP_002310709), Pt016G086400 (XP_002323456), SmIRX9 (XP_002963107), SmIRX14 (XP_002976794), Hs_GlcAT-I (AAH71961), Hs_GlcAT-P (Q9P2W7), Hs_GlcAT-S (AAL57718).

Protein sequence analysis was conducted with the software MEGA5 [20]. Multiple sequence alignments were created using the Muscle option with default settings and 500 bootstraps. Phylogenetic trees were constructed using the Neighbor-Joining method [21]. The evolutionary distances in units of the number of amino acid substitutions per site were computed using Poisson correction.

### Site-Directed Mutagenesis

Wild-type sequences of *IRX9*, *IRX9L* and *IRX14* were amplified by PCR from Arabidopsis cDNA and cloned into pDONR223 (*IRX9* and *IRX14*) and pDONRZeo (*IRX9L*) as described previously [2]. Mutated versions of *IRX9, IRX9L* and *IRX14* were generated by site-directed mutagenesis using the QuikChange Kit from Stratagene according to the manufacturer's instructions and the pDONR clones as template. Oligonucleotide primers used to introduce the various mutations are listed in Table S1. The introduction of mutations was confirmed by sequencing, performed both before and after introduction of the mutated *IRX9, IRX9L* and *IRX14* coding sequences into pEarleyGate202 destination vector [22] using LR Clonase (Life Technologies). The pEarlyGate202 vector drives expression with the cauliflower mosaic virus 35S promoter. These constructs were introduced into homozygous *irx9-2 and irx14* mutant plants and wild type Col-0 plants via *Agrobacterium*-mediated transformation using the floral dip method.

### Screening of Transformants for T-DNA Insertions and Transgenes

Transgenic plants were screened for Basta resistance and positive transformants were transferred to new pots and grown as described above. Positive transfomants were genotyped by PCR using gene-specific primers (Table S1) to verify the homozygous mutant background. To confirm the presence of the transgene in positive transformants, primer pairs annealing in the destination vector and the respective *IRX* gene were used (Table S1).

RT-PCR was used to confirm that the transgene was expressed in the selected plant lines. Total RNA was extracted from wild type and transgenic plants using the RNeasy Plant Mini Kit (QIAGEN). First-strand cDNA synthesis was performed using the SuperScript II RT Kit (Invitrogen) according to the manufacturer's protocol. RT-PCR was done with the OneStep RT-PCR Kit (QIAGEN) as described by the manufacturer using the primers listed in Table S1. *ACTIN* (*ACT2*) was used as an internal control.

### Analysis of Cell Wall Monosaccharide Composition

Alcohol-insoluble residue (AIR) of inflorescence stems was prepared and destarched enzymatically as described previously [23]. AIR samples were subsequently hydrolyzed with 2 M triflouroacetic acid (TFA) for 1 h at 120°C. Samples were dried, resuspended in water, and centrifuged for 10 min at 13,000 g. The resulting supernatant was diluted and the monosaccharide composition was determined by high-performance anion exchange chromatography (HPAEC) on a ICS 3000 instrument (Dionex) using a CarboPac™ PA20 (3×150 mm, Dionex) anion exchange column as described by Obro et al. [24].

### Xylan Immunolocalization and Microscopy

The basal regions of inflorescence stems (2.5 cm distal from the rosette) were collected from 6-week-old plants, and fixed in 4% paraformaldehyde in PEM buffer (50 mM PIPES, 5 mM EGTA, 5 mM MgSO_4_, pH 6.9) overnight at 4°C. The stems were embedded in 7% agarose and cut into 60 µm thick sections using a Leica VT1000S vibratome. Sections were incubated with the LM10 antibody (1∶10 dilution) [25], washed and subsequently incubated with rabbit anti-rat fluorescein isothiocyanate (FITC)-conjugated secondary antibodies (Sigma-Aldrich, 1∶100 dilution). Finally, sections were analyzed using a fluorescence microscope (Leica DM4000B) and the Metamorph software.

### Statistical Analysis

Quantitative growth and cell wall data were analyzed by ANOVA using SPSS v. 16.0 (IBM). Each value was expressed as means ± SD with 4–10 biological replicates. Averages were determined to be significantly different from each other (P<0.05) according to Tukey's multiple range test.

## Results

### Bioinformatic Analysis of GT43 Proteins

To identify targets for mutagenesis we first conducted a bioinformatics study of GT43 proteins. We especially focused on GlcAT-P, GlcAT-S and GlcAT-I, which are human β-1,3-glucuronosyltransferases. These proteins have been investigated in numerous site-directed mutagenesis studies and their three-dimensional structures have been resolved (see [26] for a recent review). Therefore, the key essential amino acid residues are known.

**Figure 1:**
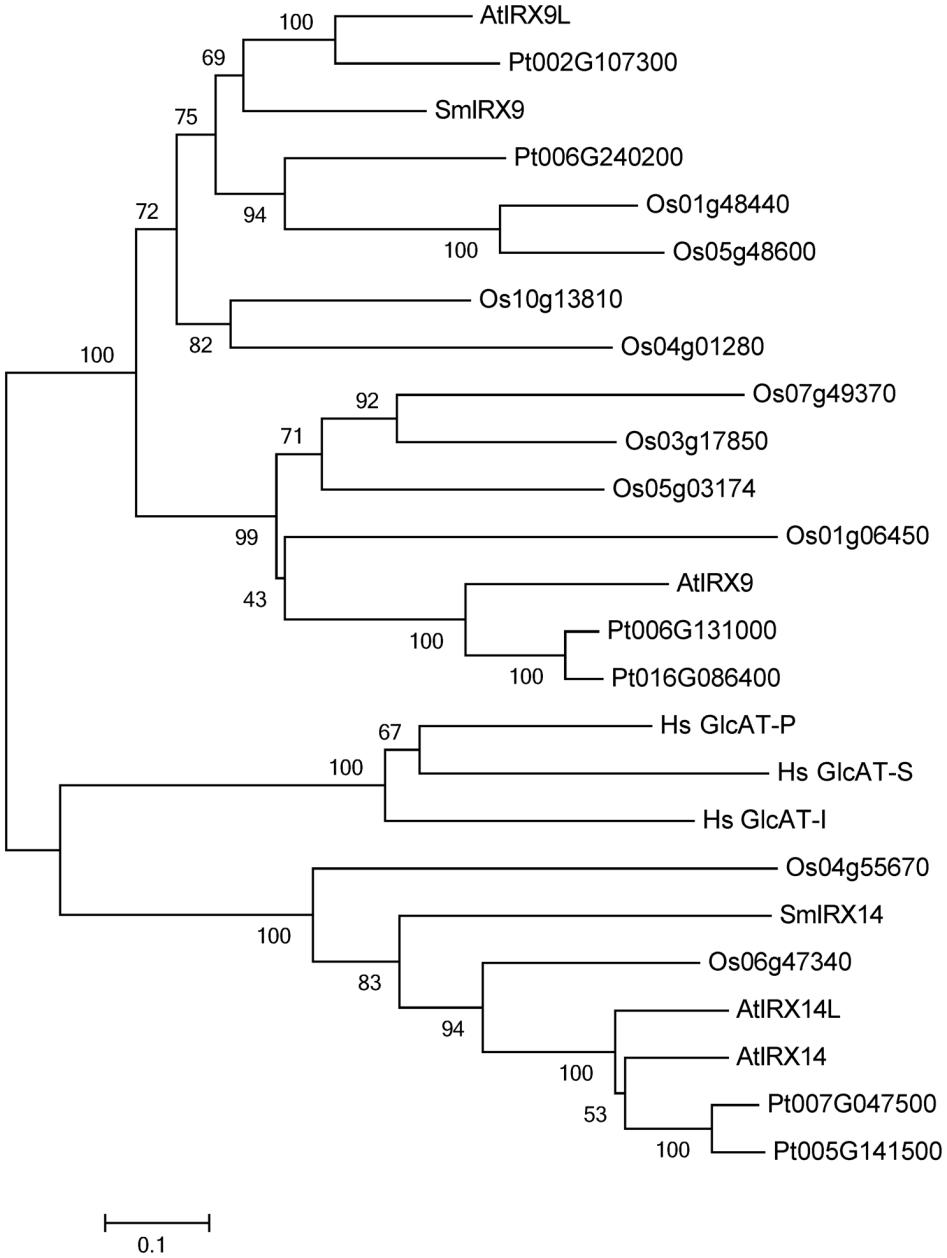
Phylogenetic tree of GT43 proteins from selected species. A multiple alignment of protein sequences from Arabidopsis (At), rice (*Oryza sativa*, Os), poplar (*Populus trichocarpa*, Pt), spikemoss (*Selaginella moellendorffii*, Sm) and human (*Homo sapiens*, Sp) was used to generate a neighbor-joining tree using MEGA5. Bootstrap values are indicated and the scale bar shows evolutionary distances in units of the number of amino acid substitutions per site. See Materials and Methods section for sequence IDs.

An alignment of GT43 members from plants and the three human GlcAT proteins is shown in Fig. S1. The phylogenetic tree shows that IRX14 and IRX14L in Arabidopsis result from a recent duplication, whereas IRX9 and IRX9L are products of a more ancient duplication in plants (Fig. 1) as also reported by Wu et al. [12]. The clade of the human GlcAT-P, GlcAT-S and GlcAT-I proteins is as closely related to IRX9 and IRX14 as the two plant clades are to each other (Fig. 1, Fig. S2).

### Site-Directed Mutagenesis

Nine different constructs were created based on conserved sequence homology and known catalytic features among GT43 proteins [27]–[29] for site-directed mutagenesis of IRX9, IRX9L and IRX14. Our sequence alignment showed the residue Trp151 of IRX9, to be 100% conserved in all the proteins analyzed. Since tryptophan has unique features in terms of chemistry and size, the conserved position could indicate its function to be important. A construct replacing tryptophan with alanine (W151A) in the IRX9 sequence was made and designated ‘IRX9-1’ (Table 1). The DxD motif, shown to be involved in donor binding and direct interaction with the metal ion required for catalysis [28] and conserved in most glycosyltransferases with a GT-A fold [27] is also observed in the GT43 proteins. In the three mammalian GlcAT proteins and IRX9L of Arabidopsis as well as in IRX9L orthologs in rice and poplar, the motif ‘DDD’ can be found. A ‘DDS’ motif in IRX14 and IRX14L was found in all species aligned, except for one rice homolog comprising a ‘DEN’ instead. However the DxD motif is known to be able to vary to some degree, e.g., comprising a glutamic acid residue instead of the aspartic acid creating motifs as ‘DxE’ or ‘ExD’. In *Selaginella*, which does not have an IRX9 ortholog, the motif is conserved as ‘DDD’ and ‘DDS’ as in the Arabidopsis IRX9L and IRX14 orthologs, respectively. Surprisingly, in the Arabidopsis and poplar IRX9 the alignment shows a conserved “GLx’ motif instead, where x is N, S or A. In rice, which has four IRX9 orthologs, two share the “GLx” motif, whereas the other two proteins have ‘AAS’ and ‘DAA’ instead (Fig. S1). Site directed mutagenesis of the DxD motif was conducted for IRX9, IRX9L and IRX14. A construct replacing the small glycine with a bulky tryptophan residue (G215W) of the IRX9 sequence was designed and designated ‘IRX9-2’ (Table 1). Furthermore the ‘GLN’ motif of IRX9 was replaced with the ‘DD(N)’ motif similar to the corresponding sequence in IRX14 (G215D/L216D, designated ‘IRX9-3’). In IRX9L the motif ‘DDD’ was changed to ‘ADA’ (D238A-D240A, designated ‘IRX9L-1’) and for IRX14 the ‘DDS’ was changed to ‘AAS’ (D261A/D262A, ‘IRX14-1’).

**Table 1:** Nomenclature used for the mutated versions of IRX9, IRX9L and IRX14.

Designation	Mutation
IRX9-0	none
IRX9-1	W151A
IRX9-2	G215W
IRX9-3	G215D/L216D
IRX9-4	E252A
IRX9-5	C323A
IRX9L-0	none
IRX9L-1	D238A-D240A
IRX14-0	none
IRX14-1	D261A/D262A
IRX14-2	Q321A
IRX14-3	C415A

We next mutagenized a Glu residue that functions as the catalytic base [28], [29]. Complete loss of catalytic activity of human GlcAT-I has been reported, when residue Glu227 was changed to Asp or Ala [29]. In the alignment all the species share a conserved Glu or Gln residue at this position (Fig. S1). Site directed mutations were made changing Glu252 to Ala in IRX9 and Gln321 to Ala in IRX14, generating ‘IRX9-4’ and ‘IRX14-2’, respectively (Table 1). Finally, the proteins have a conserved cysteine residue near the C-terminus that has proven essential for protein integrity and activity of mammalian GlcAT [30]. Changing Cys301 to Ala in GlcAT-I led to complete loss of activity. This cysteine residue is found conserved in all the plant proteins we looked at (Fig. S1), and mutations were made by changing the cysteines at this position in IRX9 and IRX14 to alanine, generating ‘IRX9-5’ and ‘IRX14-3’, respectively (Table 1).

The constructs with the mutated and wild-type versions of *IRX9* and *IRX9L* were used to transform wild-type and *irx9-2* plants, whereas the wild-type and mutated versions of *IRX14* were transformed into wild-type and *irx14* plants. Transgenic plants were identified and lines showing highest expression and maximum complementation of the mutant growth phenotype were selected for further analysis. The transformed wild-type plants served as controls to investigate if overexpression in the wild-type background would lead to increased xylan deposition or if mutated IRX proteins could have a dominant negative effect.

### Analysis of Transformants

Several independent transformants (n = 2−10) were selected for each construct in the T1 generation. The most clearly complementing line was selected for each construct and further analyzed in T2. For the IRX14-1 construct, six independent T1 lines were obtained, none of which showed substantial complementation, despite expressing the transgene. Also for this construct, the line that showed most complementation was selected for analysis in T2. All further analysis reported below was done with the T2 plants.

**Figure 2:**
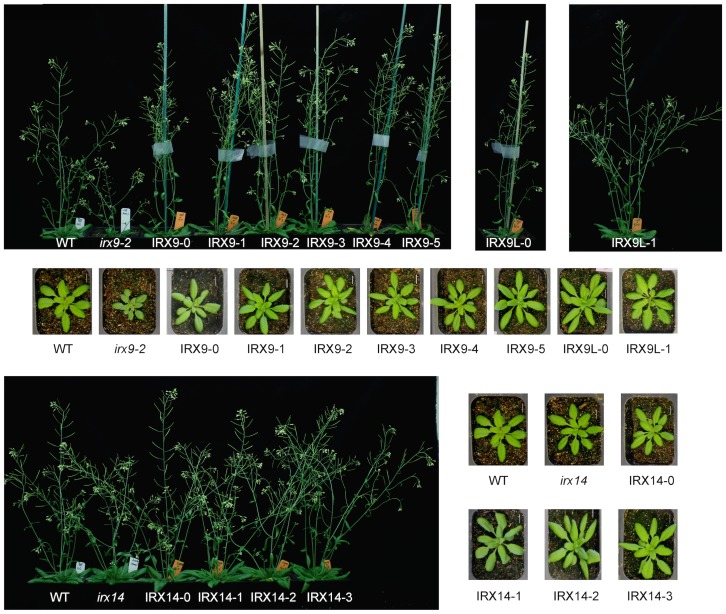
Representative photos of wild type, mutants and transformed plants. The nomenclature for the constructs used to transform the *irx9* and *irx14* plants is explained in Table 1. Rosettes were from 4-week-old plants and mature plants were 6-week-old.

**Figure 3:**
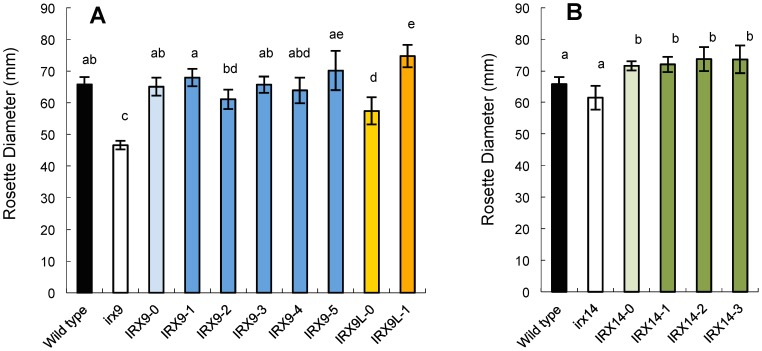
Rosette diameter of 4-week-old mutant plants transformed with the different constructs. The nomenclature for the constructs used to transform the *irx9* and *irx14* plants is explained in Table 1. The bars show average ± SD (n = 6). Averages that are not significantly different (ANOVA, Tukey's test, p>0.05) are indicated with the same letter.

**Figure 4:**
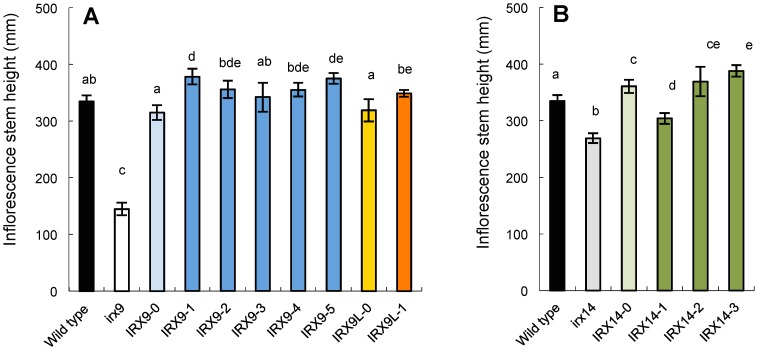
Stem height of 6-week-old mutant plants transformed with the different constructs. The nomenclature for the constructs used to transform the *irx9* and *irx14* plants is explained in Table 1. The bars show average ± SD (n = 6). Averages that are not significantly different (ANOVA, Tukey's test, p>0.05) are indicated with the same letter.

Representative photos of the selected transgenic T2 plants are shown in Fig. 2. Generally, it was obvious that all the IRX9 and IRX9L constructs complemented the growth phenotype of *irx9-2*. The *irx14* mutant does not have a strong growth phenotype so it was not immediately obvious to what extent the IRX14 constructs complemented the phenotype. The level of complementation was quantified in several ways. Firstly, the rosette diameter (Fig. 3) and stem height were determined (Fig. 4). These results confirmed that the wild-type and all mutated versions of IRX9 and IRX9L complemented the *irx9* growth phenotype. The wild-type and mutated IRX14 proteins all complemented the *irx14* mutation, except for IRX14-1 where the ‘DDS’ motif was changed to ‘AAS’. The IRX14-1 construct only partially complemented the growth phenotype as the stem height was intermediary between the *irx14* mutant and the wild type. None of the mutated proteins had a dominant negative effect in the wild-type background – this could have been expected with the IRX14-1 protein if the mutated protein would ‘poison’ a protein complex, but we did not observe that (Figs. S3–S5).

**Figure 5:**
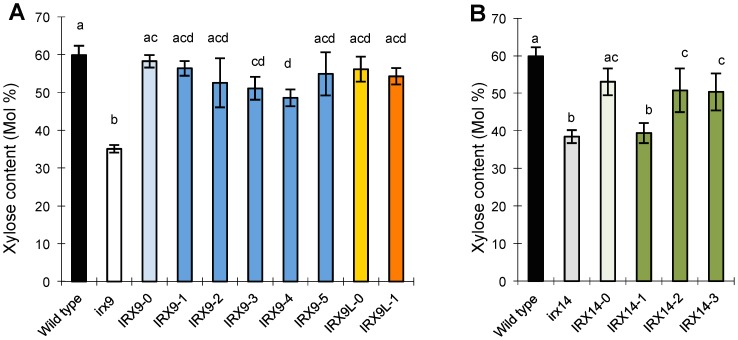
Xylose content in cell wall polysaccharides isolated from stems of 6-week-old mutant plants transformed with the different constructs. Cell walls were prepared from stems, hydrolyzed in TFA, and the xylose content determined by HPAEC. The nomenclature for the constructs used to transform the *irx9* and *irx14* plants is explained in Table 1. The bars show average ± SD (n = 4). Averages that are not significantly different (ANOVA, Tukey's test, p>0.05) are indicated with the same letter.

We next analyzed the sugar composition in the stems of T2 plants. Especially for IRX14 biochemical analysis of the phenotype is more accurate since the growth phenotype of the *irx14* mutant is mild. All wild-type and mutant versions of IRX9, IRX9L and IRX14 complemented the decreased xylose phenotypes of the *irx9* and *irx14* mutants, again with the notable exception of the IRX14-1 plants, for which the xylose content resembled mutant levels (Fig. 5). In some cases the complementation was not completely to the wild-type level, but the ability of the mutated proteins to complement also did not differ from the complementation seen with wild-type versions of the proteins – except for IRX14-1.

**Figure 6:**
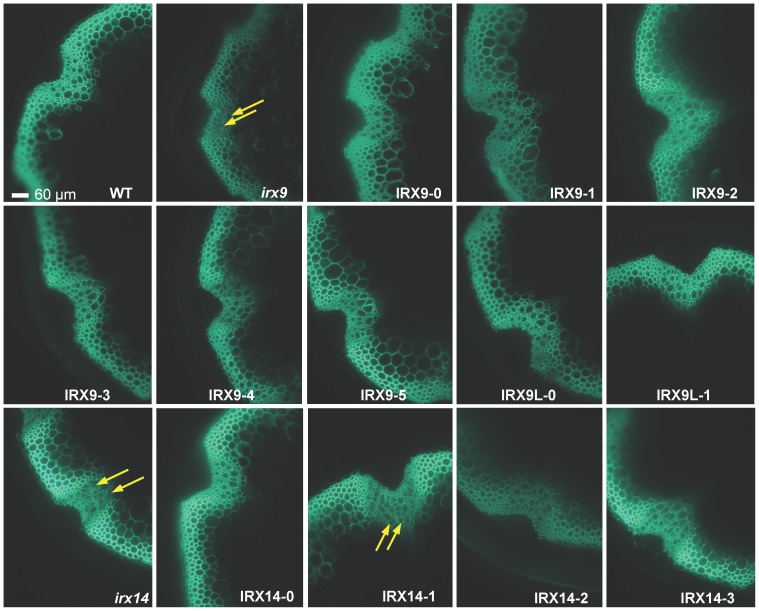
Microcopy analysis of stems from 6-week-old plants. The LM10 anti-xylan monoclonal antibody was used for immunodetection of xylan in transverse stem sections. The nomenclature for the constructs used to transform the *irx9* and *irx14* plants is explained in Table 1. Irregular xylem phenotype (indicated with arrows) was observed in the *irx9* and *irx14* mutants, as well as in the *irx14* plants transformed with the 35S:IRX14-1 construct. All the other transformants showed normal vessel phenotype.

Finally, we investigated the irregular xylem phenotype in stem cross sections from T2 plants (Fig. 6). Irregular xylem (irx) phenotype was observed in the *irx9* and *irx14* as expected, and consistent with the other data, the irx phenotype was complemented in all the transformed plant lines, except for IRX14-1.

## Discussion

This study shows that amino acid residues that have been shown to be essential in mammalian GT43 proteins are not important for the biological function of the Arabidopsis IRX9 and IRX9L proteins. All mutated versions of the proteins showed complementation of the *irx9* phenotype. This result is in agreement with the partial function of a truncated IRX9 protein in the *irx9-2* mutant, which is clearly too short to have catalytic glycosyltransferase function, but still plays a significant role as the growth phenotype is much milder than in *irx9* knock-out plants. Our observations and the phenotype of *irx9-2* strongly indicate that IRX9 and IRX9L have important non-catalytic functions. The absence of the DxD motif in IRX9 is surprising but also suggests that the protein is not catalytically active. Nevertheless, in the absence of an in vitro assay to test the activity of these proteins it is not possible to exclude that they possess glycosyltransferase activity. For IRX14 the results are more ambiguous since the DxD motif is clearly important for complementation, whereas the Gln residue in the predicted acceptor-binding and catalytic site is not. In the well-characterized human proteins the corresponding catalytic residue is actually Glu and not Gln. However, whether Gln and Glu could have the same function is not clear. Hence, we speculate that the UDP-xylose binding of IRX14 is important for xylan synthesis but that it does not essentially have to participate in xylosyl transfer. A conceivable scenario is that IRX14 passes the UDP-xylose substrate on to another protein, most likely IRX10 and IRX10L. However that raises the question, what is the role of IRX9 and IRX9L? We think our data indicate that IRX9 and IRX9L have a structural role, most likely helping to organize and/or assemble a protein complex, which is necessary for proper xylosyltransferase function in Arabidopsis. IRX10 and IRX10L are the likely catalytic subunits in such a complex. This is reminiscent of GAUT7, which is known to be catalytically inactive, despite being a member of the GAUT family of galacturonosyltransferases. The role of GAUT7 is instead the anchoring of GAUT1 in the Golgi, and thus GAUT7 is essential for the catalytical function of GAUT1 and ultimately for homogalacturonan synthesis despite not being a galacturonosyltransferase itself [31]. The interactions between IRX9, IRX14 and IRX10 have been difficult to show but can also not be excluded. Presumably interactions between these proteins are not very strong, which makes it difficult to prove them. Also protein tags that have been used to investigate interactions might interfere and thereby prohibit interactions. A study in wheat showed coimmunoprecipitation of IRX14 and IRX10, but IRX9 homologs were missing from the preparation and a direct interaction between the IRX14 and IRX10 homologs was not demonstrated [16].

Expression of the non-functional IRX14-1 protein in the wild-type background had no effect. In many cases, non-functional proteins that are part of a protein complex have a dominant negative effect on enzyme activity because the non-functional subunit interacts with the other subunits in the complex and prevent their normal function. However, we did not observe such an effect, perhaps because the normal IRX14 protein interacts more strongly or because the expression level of IRX14-1 was insufficient to bind a large proportion of the IRX10/IRX10L proteins. This could suggest that loss-of-function mutations in IRX10 or IRX10L would be more likely to have a dominant negative effect on xylan synthesis.

## Supporting Information

Figure S1 **Multiple sequence alignment of GT43 protein sequences.** Multiple sequence alignment was created with the Muscle option in MEGA5. The N- and C-terminal regions that aligned less well are omitted in the figure. All GT43 proteins encoded in the genomes of Arabidopsis (At), rice (*Oryza sativa*, Os), poplar (*Populus trichocarpa*, Pt), spikemoss (*Selaginella moellendorffii*, Sm) and human (*Homo sapiens*, Sp) were included in the analysis. Conserved regions that were analyzed by site-directed mutagenesis are marked with blue, and the actual residues that were altered are marked with yellow. The changes made in the mutated proteins are listed in Table 1.
(PDF)

Figure S2 **Identity matrix for protein sequences used to make the alignment is shown in Fig. S1 and the phylogenetic tree in** **Fig. 1** **.**
(TIFF)

Figure S3 **Rosette diameter of 4-week-old wild-type plants transformed with the different constructs.** The nomenclature for the constructs used to transform the plants is explained in Table 1. Wild type, *irx9*, and *irx14* were included for comparison. The bars show average ± SD (n = 10). Averages that are not significantly different (ANOVA, Tukey's test, p>0.05) are indicated with the same letter.
(TIFF)

Figure S4 **Stem height of 6-week-old wild-type plants transformed with the different constructs.** The nomenclature for the constructs used to transform the plants is explained in Table 1. Wild type, *irx9*, and *irx14* were included for comparison. The bars show average ± SD (n = 10). Averages that are not significantly different (ANOVA, Tukey's test, p>0.05) are indicated with the same letter.
(TIFF)

Figure S5 **Xylose content in cell wall polysaccharides isolated from stems of 6-week-old wild-type plants transformed with the different constructs.** Cell walls were prepared from stems, hydrolyzed in TFA, and the xylose content determined by HPAEC. The nomenclature for the constructs used to transform the plants is explained in Table 1. Wild type, *irx9*, and *irx14* were included for comparison. The bars show average ± SD (n = 4,except for WT+IRX9-4 transformants, where n = 3). Averages that are not significantly different (ANOVA, Tukey's test, p>0.05) are indicated with the same letter.
(TIFF)

Table S1 Primers used for cloning, genotyping and RT-PCR.
(PDF)
