# Supplementary material for: Site-Directed Mutagenesis of IRX9, IRX9L and IRX14 Proteins Involved in Xylan Biosynthesis: Glycosyltransferase Activity Is Not Required for IRX9 Function in Arabidopsis
Source: PLoS One. 2014 Aug 13;9(8):e105014. doi: 10.1371/journal.pone.0105014 (PMC4132061; doi:10.1371/journal.pone.0105014)
Supplement: Figure S1 — Multiple sequence alignment of GT43 protein sequences. Multiple sequence alignment was created with the Muscle option in MEGA5. The N- and C-terminal regions that aligned less well are omitted in the figure. All GT43 proteins encoded in the genomes of Arabidopsis (At), rice (Oryza sativa, Os), poplar (Populus trichocarpa, Pt), spikemoss (Selaginella moellendorffii, Sm) and human (Homo sapiens, Sp) were included in the analysis. Conserved regions that were analyzed by site-directed mutagenesis are marked with blue, and the actual residues that were altered are marked with yellow. The changes made in the mutated proteins are listed in Table 1. (PDF) [file pone.0105014.s001.pdf]

```
AtIRX9            ATYTQHSLLNRTLINSQSQA----PAPAESREAEGETRSLSEKED-ENQVKVTPRGLVIV 119
Pt006G131000      VASTPHASNVNRSLIAESPV----PTPLSSKESEPAKFLEKEEEP---KPKLLPRRLAII 114
Pt016G086400      AASTPHASNVNRSLIAETAV----PAPPSSKESEHATFLGKEETE----SKLAPRRLAII 118
Os01g06450        ----------------------------ATSIDSIRASFRPTVAAT----PPVPELDLLLI 69
Os03g17850        ------------------SH----VVRSLHATGGAAVNRSLLAQAAAGAVDAGPQPLLVV 100
Os05g03174        ----RHQLAASHVAVNQQVS----LVPDAAAAEAAGVGNGAVVDVGDDEGGEGARRMLIV 112
Os07g49370        ----------------DWPAAAGAAVFLRTLRASNVIFSRSSNRP--------QQPQLVV 84
AtIRX9L           ---QPYVEERLENRKRE-------------EAAVDAVSFVAETENGKKEVNFVPKKLLIV 145
Pt002G107300      -----------PHVNVQLDT----KDNFALAAVSLGVEKTTPQLDRFSRFDYVERKQVIV 145
SmIRX9            FTAAKRIATEEQVVVLDSSS----PLEISANSSRHPGEGRMPLQD--------PKKLLII 109
Pt006G240200      TRNATTIAERGGLENSTALE----PQVKEEESGDGNSNGTSISLSLSEDVNLVSRKLLII 193
Os01g48440        TKLEPFVAEAESEASSEPQVEEGPPVPAMLDDEADFVEASPIVHS-VNDSGIVVRKHLII 197
Os05g48600        KELETIVVEKEVDIIDESEVEESPPVPAMLDDEADFVESAPAIPD-INDLDITVRKLLII 199
Os10g13810        ------------------------PATAILVREDPPSVVVDVDTP--LPAAAEERKLLLV 81
Os04g01280        -----------HLPRPLGPI----PNPNSHHRHRDPFPILQHPHP--PSTPHSNHKLLIV 130
AtIRX14           TKSSRVVVGRHGIRIRPWPH----PNPVEVMKAHQIIGRVQKEQK--MIFGMKSSKMVIA 164
AtIRX14L          KKNSRVVVGRHGIRIRPWPH----PNPIEVLRAHQLLVRVQKEQK--SMYGVRSPRTVIV 156
Pt007G047500      TVSSRVVVGRHGIRIRPWPH----PNPSEVIKAHQIIERVQREQS--NQFGVKSPRSLIV 161
Pt005G141500      SSSSRVVVGRHGIRIRPWPH----PNPSEVMKAHQIIETVQREQR--TQFGVKSPRTLIV 160
Os06g47340        HTQSHVVVGRHGIRIRPWPH----PDPVEVMRAHRIMERVQEEQR--RWYGVKEPRHVLV 200
Os04g55670        HHHGPVFVGRHPIRVRPWPH----PDPNELLKAHHILAAVQNAQRSSRRRGAGPPRPVIA 174
SmIRX14           TKSGRVHVGRHEILIRPWPH----PDSAEIYRAHALLERVQVEQQ--SLYGPKERKTVIA 165
Hs_GlcAT-I        DCLPPLRAAAEQLRQKDLRI----SQLQAELRRPPPAPAQPPEPE--------ALPTIYV 79
Hs_GlcAT-P        GADPREYCTSDRDIV-------------EVVRTEYVYTRPPPWSD--------TLPTIHV 88
Hs_GlcAT-S        PYFSPYAVGRGGARLP-------------LRRGGPAHGTQKRNQSRPQPQPEPQLPTIYA 84

AtIRX9            VTPIITKDR-----YKNVLLRRMANTLRLV-PPPLLWIVVEKHS----DGEEKSSSTMLR 169
Pt006G131000      VTPISTEDP-----YQGVFLRRLANTIRLV-PPPLLWIVVEGQS----DSDE--VSEILR 162
Pt016G086400      VTPTSTKDP-----YQGVFLRRLANTIRLV-PPPLLWIVVEGQS----DSDE--VSEVLR 166
Os01g06450        VTVTRPDDDDDDGMSQEASLTRLGHTLRLV-EPPLLWIVVGAEN----TTATARAVNALR 124
Os03g17850        VTTTESTPSAAG--QRAAALTRMAHTLRLV-PPPLLWVVVEANP----DVAA--TARLLR 151
Os05g03174        VTTTRGERR-----RRRGELLRLAHTLRLV-RPPVVWVVVEPAA----DAAA--TAEVLR 160
Os07g49370        VVTTTEQSDDSE--RRAAGLTRTAHALRLV-SPPLLWLVVEEAPAEKHAAPP--TARLLR 139
AtIRX9L           VTPTYNRA------MQAYYLNRVAQTLRLV-ESPVLWIVVEGNV----ASFE--TSEILR 192
Pt002G107300      ITPTYNRA------LQAYFLNRLGQVLRLV-QPPLLWIVVEMTS----ASAE--TAEILR 192
SmIRX9            VTPTYTRP------FQAMYLTRLAHTLKLV-DPPLLWIVVEMPG----QSLE--TASLLR 156
Pt006G240200      VTPTHARP------LQAYYLSRLAHTLKLV-QPPLLWIVVEMTL----QSDH--TADILR 240
Os01g48440        ITTTSVRP------HQAYYLNRLAHVLKDV-PPPLLWIVAEWPY----QSRE--TAEILR 244
Os05g48600        VTITTVRP------QQAYYLNRLAHVLKTV-QSPLLWLVVEWPD----QSFQ--TAEILR 246
Os10g13810        VTPTRARP------LQAYYLRRLAHTLRLA-PSPLLWLVVESGA----ATRD--TAALLR 128
Os04g01280        VTPTRARP------SQAYYLTRMAHTLRLLHDSPLLWIVVQAGN----PTPE--AAAALR 178
AtIRX14           VTPTYVRT------FQALHLTGVMHSLMLV-PYDLVWIVVEAGG----ATNE--TGLIIA 211
AtIRX14L          VTPTYVRT------FQALHLTGVMHSLMLV-PYDLVWIVVEAGG----ITNE--TASFIA 203
Pt007G047500      VTPTYVRT------FQTLHMTGVMHSLMLL-PYDVVWIVVEAGG----VTNE--TALIIA 208
Pt005G141500      VTPTYVRT------FQTLHLTGVMHSLMLV-PYDVVWIVVEAGG----ATNE--TASIIA 207
Os06g47340        VTPTYSRA------FQALHLTGLLHSLRNV-PYPLTWIVVEAGG----TTNA--TASLLA 247
Os04g55670        VTPTTTSA------LQVPSLTSMAHTLRLV-DGPLTWIVVEPEH----HTDA--VAAVLS 221
SmIRX14           ITPTFART------FQAIHLLGVMHTLRAA-PGPVIWIVVEAGG----RSNE--TASILA 212
Hs_GlcAT-I        VTPTYARL------VQKAELVRLSQTLSLV--PRLHWLLVEDA-----EGPTPLVSGLLA 126
Hs_GlcAT-P        VTPTYSRP------VQKAELTRMANTLLHV--PNLHWLVVEDAP-----RRTPLTARLLR 135
Hs_GlcAT-S        ITPTYSRP------VQKAELTRLANTFRQV--AQLHWILVEDAA----ARSE-LVSRFLA 131
                  :.             :   :    : :       : *::.                  :
```

```
AtIRX9                  KTGIMYRRIVF--K---EDFTSLES----E-----LDHQRNLALRHIE------------ 203
Pt006G131000            KTGIMYRHLVI--K---ENFTDPEA----E-----LDHQRNVALRHIE------------ 196
Pt016G086400            KTGIMYRHLVF--K---ENFTDPEA----E-----LDHQRNVALRHIE------------ 200
Os01g06450              GTRVMFRHLTY--AA--ENFTGPAGD---E-----VDYQMNVALSHIQ------------ 160
Os03g17850              TTGLMYRHLTY--K---DNFTVADAAAGKE-----RHHQRNVALGHIE------------ 189
Os05g03174              GTGVMYRHLAF--RP-EENFTTADA----E-----AHAQRNAALAHVE------------ 196
Os07g49370              RTGVVHRHLLM--KQGDDDFSMQISM---R-----REQQRNVALRHIE------------ 177
AtIRX9L                 KTGVMYRHLVC--K---RNMTSIKD----R-----GVHQRNTALEHIE------------ 226
Pt002G107300            KTGVMYRHLVC-VN---KNNTNVKD----R-----GVHQRNAGLEHIE------------ 227
SmIRX9                  KTGVMYRHLAC--E---KNLTNVKD----R-----GTQQRNLALQHIE------------ 190
Pt006G240200            RTGVMYRHLVC--N---KNLTDIKD----R-----SVHQRNVALSHIE------------ 274
Os01g48440              SSGIMYRHLIC--N---RNTTNIRK----I-----VVCQKNNAIFHIK------------ 278
Os05g48600              SSGVMYRHLIC--R---KNTTSVRK----I-----AVCQRNTAIYHIK------------ 280
Os10g13810              GCGVMYRHLSSPVPDAPQDRPRRRGRRQDRPAVDSRARQRNTALDHIE------------ 176
Os04g01280              RTAVLHRYVGCCHN---INASAPDF----------RPHQINAALDIVD------------ 213
AtIRX14                 KSGLRTIHVGI--D---QRMPNTWEDRSKL-----EVFMRLQALRVVR------------ 249
AtIRX14L                KSGLKTIHLGF--D---QKMPNTWEDRHKL-----ETKMRLHALRVVR------------ 241
Pt007G047500            KSGVKTLHIGF--N---QKMPNSWEGRHRL-----ETKMRLRALRVVR------------ 246
Pt005G141500            KSSIKTFHIGF--T---QKMPNSWEGRHKL-----ETKMRLRALRVVR------------ 245
Os06g47340              RSDLTIVHIPF--P---DRMPHDWADRHAT-----ENRMRLHALRVIR------------ 285
Os04g55670              RSNLNFLHI-----------TGPDS----S-----TSRLRMHALREIR------------ 249
SmIRX14                 SSRLEFVHLGV--K---DAMPVAWEQRRRM-----ETRLRIEGLSHVR------------ 250
Hs_GlcAT-I              ASGLLFTHLVVLTPKAQRLREGEPGWVHPR-----GVEQRNKALDWLRGRGGAVGGEKDP 181
Hs_GlcAT-P              DTGLNYTHLHVETPRNYKLRGDARDPRIPR-----GTMQRNLALRWLR--------ETFP 182
Hs_GlcAT-S              RAGLPSTHLHVPTP---RRYKRPGLPR--------ATEQRNAGLAWLR--------QRHQ 172
                           :    :                                 .:  :

AtIRX9                  -HHKLSGIVHFAGLNNIYDLDFFVKIRDIEVFGTWPMALLSANRK--------------- 247
Pt006G131000            -QHRLSGIVHFAGLSNVYDLGFFDELRQIEVFGTWPVALLSANKN--------------- 240
Pt016G086400            -KHRLSGIVHFAGLSNVYDLGFFDEIRQIEVFGTWPMALLSANEK--------------- 244
Os01g06450              -LHRLPGVVHFAAASSVYDLRFFQQLRQTRGIAAWPIATVSSADQ--------------- 204
Os03g17850              -HHRLAGVVLFAGLGDTFDLRFFDQLRQIRTFGAWPVATMSQNER--------------- 233
Os05g03174              -KHRLSGVVHFADAAGVYDAHFFDEIRQIEAFGTWPVATMSAGEK--------------- 240
Os07g49370              -DHRIAGVVLFGGLADIYDLRLLHHLRDIRTFGAWPVATVSAYER--------------- 221
AtIRX9L                 -LHKLDGIVYFADDDNIYSLELFQSLRQISRFGTWPVAMLAQSKN--------------- 270
Pt002G107300            -RHRLDGIVYFADDDNVYSLQLFESLRNISHFGTWPVAMLAQSKN--------------- 271
SmIRX9                  -RHQLDGIVYFADDDNFYSLELFDQLREIKRFGTWPVAMLAHSKS--------------- 234
Pt006G240200            -IHHLDGIVHFADDYNTYSADLFEQMRQIRRFGTWTVAKLTGNKN--------------- 318
Os01g48440              -KHRLDGIVHFADEERAYSADLFEEMRKIRRFGTWPVAIHVGTKY--------------- 322
Os05g48600              -KHRLDGIMHFADEERSYMSDVFEEMRKIRRFGAWPVAIHTGIKY--------------- 324
Os10g13810              -HHRLHGIVYFADEDNVYSLDLFYHLRDIRSFGTWPVATLAPGKS--------------- 220
Os04g01280              -NHRLDGVLYFADEEGVYSLHLFHHLRQIRRFATWPVPEISQHTN--------------- 257
AtIRX14                 -EEKLDGIVMFADDSNMHSMELFDEIQNVKWFGTVSVGILAHSGNAEEMVLS-MEKRKEM 307
AtIRX14L                -EKKLDGIVMFADDSNMHSMELFDEIQTVKWFGALSVGILAHSGNADE-----LSSILKN 295
Pt007G047500            -EEKMDGIVMFADDSNMHSMELFDEIQNVKWFGAVSVGILVHSGGADETLLTAAAMVDK 305
Pt005G141500            -EEMMDGIVMFADDSNMHSMELFDEIQNVKWFGAVSVGILAHSGGGGES----SSAVAEK 300
Os06g47340              -ERKMDGVIVFADDSNVHSLELFDEVQKVQWMGAVSVGILAHTGTADQPRLS-------- 336
Os04g55670              -KRKMDGVVVFADENSILRTELFDEAQKVKSVGAVPVGVLGEDEG--------------- 293
SmIRX14                 -REKLDGLILFTDDSNVHSLQLFDEIQKVKWIGALSVGLLETGSGATETASS-------M 302
Hs_GlcAT-I              PPPGTQGVVYFADDDNTYSRELFEEMRWTRGVSVWPVGLVGGLRF--------------- 226
Hs_GlcAT-P              RNSSQPGVVYFADDDNTYSLELFEEMRSTRRVSVWPVAFVGGLRY--------------- 227
Hs_GlcAT-S              HQRAQPGVLFFADDDNTYSLELFQEMRTTRKVSVWPVGLVGGRRY--------------- 217
                              *:: *          .:   :    ... .:
```

```
AtIRX9            ---------RVVVEGPVC--ES-SQVL--GWHLR------------------------KI 269
Pt006G131000      ---------KVTIEGPVC--DS-SQVI--GWHLK------------------------KM 262
Pt016G086400      ---------KVIIEGPVC--DS-SQVI--GWHLR------------------------KM 266
Os01g06450        ---------TVKLEGPTC--NS-SQIT--GWYSKDSSSNITETTWDSSSNTTQTTWDSSS 250
Os03g17850        ---------KVVVQGPAC--SS-SSVA--GWFSM------------------------DL 255
Os05g03174        ---------KVVVEGPLC--SD-SKVV--GWFSR----------------------DFND 264
Os07g49370        ---------KVMVQGPLCINTSSSSVITRGWFDM------------------------DM 248
AtIRX9L           ---------KAILEGPVC--NG-SQVI--GWHT-------------------------NE 291
Pt002G107300      ---------KAIVEGPVC--NA-SQVI--GWHT-------------------------NE 292
SmIRX9            ---------KTILEGPVC--DG-HKVT--GWHT-------------------------NE 255
Pt006G240200      ---------KDFVEGPIC--NG-TQVI--GWHV-------------------------ND 339
Os01g48440        ---------RVVLEGPVC--KG-NQVT--GWHTN------------------------QR 344
Os05g48600        ---------RVVLEGPIC--KG-NRVT--GWNTI------------------------QN 346
Os10g13810        ---------KTILQGPVC--EG-SRVV--GWHTT------------------------DR 242
Os04g01280        ---------EVVLQGPVC--KQ-GQVV--GWHTT------------------------HD 279
AtIRX14           EKEEEEESSSLPVQGPAC--NSTDQLI--GWHIF------------------------NT 339
AtIRX14L          EQGKNKEKPSMPIQGPSC--NSSEKLV--GWHIF------------------------NT 327
Pt007G047500      EAEENLPNPVVPVQGPAC--NASNKLV--GWHTF------------------------NS 337
Pt005G141500      DVKPNLSNPAMPVQGPAC--NASNKLV--GWHTF------------------------NS 332
Os06g47340        --EEDKQNMPLPVQGPAC--NSSGHLA--GWHTF------------------------NS 366
Os04g55670        -------TSETFLQAPSC--DAEGKLV--GYHVS------------------------EE 318
SmIRX14           VAAASSAKPRLPVQGPAC--NETCHVV--GWHVL------------------------RP 334
Hs_GlcAT-I        -------------EGPQV--QD-GRVV--GFHTA-------------------------- 242
Hs_GlcAT-P        -------------EAPRV--NGAGKVV--GWKTV-------------------------- 244
Hs_GlcAT-S        -------------ERPLV--EN-GKVV--GWYTG-------------------------- 233
                               : *         :   *:

AtIRX9            N----------------NETETKPP---IHISSFAFNSSILWDPERWG------------ 298
Pt006G131000      N----------------NETDKRPP---IHISSFGFNSSILWDPERWG------------ 291
Pt016G086400      N----------------NETDKRPP---IHISSFGFNSSILWDPERWG------------ 295
Os01g06450        NKTQTTTLAALDTNASKQNSSSGPPE--INMHAVGFKSSMLWDSERFT------------ 296
Os03g17850        SNATSPVAVGGAGY---GAAAARPRE--LDVHGFAFNSSVLWDPERWG------------ 298
Os05g03174        GTTRAVTYNTEADLNPAGAAGTRAHT--IDVSGFAFNSSILWDPERWG------------ 310
Os07g49370        DMAAGGERR--------AAADRPPPETLMEVGGFAFSSWMLWDPHRWD------------ 288
AtIRX9L           K----------------SKRLRRFH---VDMSGFAFNSTILWDPKRWR------------ 320
Pt002G107300      K----------------SKRLRRFH---VDMSGFAFNSTILWDPKRWN------------ 321
SmIRX9            K----------------SKRLRRFH---VDMSGFGFNSTILWDPRRWK------------ 284
Pt006G240200      S----------------RRRFRRFH---ADMSGFAFNSTIIWDPKRWH------------ 368
Os01g48440        -----------------RGVSRRFP---IGFSGFAFNSTILWDPQRWN------------ 372
Os05g48600        IQK--------------KSAVRRFP---VGFSGFAFNSTMLWDPERWN------------ 377
Os10g13810        -----------------SKNQRRFH---VDMSGFAFNSSKLWDAKNRG------------ 270
Os04g01280        -----------------GNKLRRFH---LAMSGFAFNSTMLWDPKLRS------------ 307
AtIRX14           LPYAGKSAVYID-----DVAAVLPQK--LEWSGFVLNSRLLWEEA-EN------------ 379
AtIRX14L          QPYAKKTAVYID-----EKAPVMPSK--MEWSGFVLNSRLLWKESLDD------------ 368
Pt007G047500      LPYEGKSAVYID-----DRATVLPRK--LEWAGFMLNSRLLWKEA-ED------------ 377
Pt005G141500      LPYEGKSAVYID-----DRATVLPRK--LEWAGFVLNSRLLLKEA-QD------------ 372
Os06g47340        LPFAGKTATVVG-----EAAPVLPRG--LEWAGFVLNSRILWKEA-EG------------ 406
Os04g55670        TMLPANR----------GDMLLSSR---LEWAGFVVNAQALWEGGGAAS----------- 354
SmIRX14           SPVDGEDDSSSSFTDVAGGLTDVATH--LEWSGFVINSRAVWDEA-ESENEEEEASSSTM 391
Hs_GlcAT-I        -----------------WEPSRPFP---VDMAGFAVALPLLLD----------------- 265
Hs_GlcAT-P        -----------------FDPHRPFA---IDMAGFAVNLRLILQ----------------- 267
Hs_GlcAT-S        -----------------WRADRPFA---IDMAGFAVSLQVILS----------------- 256
                                                  .. .    : .
```

```
AtIRX9            -----------------R-PSSVEGTKQDSIK-------YVKQVVLED--DTKLKGLPAQD 332
Pt006G131000      -----------------R-PSSVQQTSQNSIK-------FVKQAALED--ETELKGIPPED 325
Pt016G086400      -----------------R-PSSVQQTSQNSIK-------FVKQVALED--ETKLKGIPPED 329
Os01g06450        -----------------RRDNSSTGINQDLIQ-------AVRQMMIND--EDKKRGIP-SD 330
Os03g17850        -----------------RYPTSEPDKSQDSVK-------FVQQVVLED--YSKVRGIP-SD 332
Os05g03174        -----------------R-PTSLPDTSQDSIK-------FVQEVVLED--RTKLKGIP-SD 343
Os07g49370        -----------------RFPLSDPDASQESVK-------FVQRVAVEEYNQSTTRGMPDSD 325
AtIRX9L           -----------------R-PFSHPTRQLDTVKEGFQETSFIEQV-VAD--ESEMEGVP-PA 359
Pt002G107300      -----------------R-PFSNPIRQLDTVKEGFQETTFIEQV-VED--ESQMESVP-PS 360
SmIRX9            -----------------R-PTTQPVRQLDTVKEGFQETTFIEQL-VED--ENQMEGLP-SG 323
Pt006G240200      -----------------R-PTPEPIRQLDTVRDGFQVSSFIEQV-VED--ESQMEGLL-ED 407
Os01g48440        -----------------S-PTLESIIVHSGGRGGLQESRFIEKL-VED--ESQMEGLG-DN 411
Os05g48600        -----------------R-PPMDSVIVHSGGRGGLQESRFIEKL-VKH--ERQIEGLP-ED 416
Os10g13810        -----------------H-QAWNYIRQLDTAKEGFQETAFIEQL-VED--ETHMEGVP-PG 309
Os04g01280        -----------------H-LAWNSIRHPEMVKESLQGSAFVEQL-VED--ESQMEGIP-AD 346
AtIRX14           -----------------K-PEWVKDF--GSLNEN-EGVESPLSL-LKD--PSMVEPLG--S 414
AtIRX14L          -----------------K-PAWVKDL--SLLDDGYAEIESPLSL-VKD--PSMVEPLG--S 404
Pt007G047500      -----------------K-PEWVKDM--DLVDEN---IENPLAL-LKD--PSMVEPLG--S 410
Pt005G141500      -----------------K-PEWVKDL--DLVDEN---IESPLAL-LKD--PSMVEPLG--S 405
Os06g47340        -----------------K-PDWVKDL--DAVGENGEEIENPLIL-LND--PSSVEPLG--N 442
Os04g55670        -----------------R-PEWVSDI--DAIDDG--AAASPLSL-VTD--AARVEPLA--S 388
SmIRX14           EDEQQEEDAERASTRRK-PEWINEWK-EWISED---SGSPLRL-AKD--QGFIEALG--N 441
Hs_GlcAT-I        -----------------K-PNAQFD---STAPRGHLESSLLSHL--VD--PKDLEPRA-AN 300
Hs_GlcAT-P        -----------------R-SQAYFKL--RGVKGGYQESSLLRELVTLN--D--LEPKA-AN 303
Hs_GlcAT-S        -----------------N-PKAVFKR--RGSQPGMQESDFLKQITTVE--E--LEPKA-NN 292
                                                              .      .

AtIRX9            C--SKIML------------WRLKFP-------------------------------TRT 347
Pt006G131000      C--SKIML------------WRLNLP---------------------------------V 338
Pt016G086400      C--SKIML------------WRLNLP---------------------------------T 342
Os01g06450        CSDSQIML------------WHLDMPRHTPKIEQATPEKESLTKGDEEESHDMTLDNVVP 378
Os03g17850        C--SEVMAKLRTVSQQLEATWRSALA------------IINELLRACASVHGHVRSKLDS 378
Os05g03174        C--SQIMV------------WQYTMP---------------------------------- 355
Os07g49370        C--SQIML------------WRIQTTL--------------------------------- 338
AtIRX9L           C--SSILN------------WHLHLD------------------------------ALDV 375
Pt002G107300      C--SRILN------------WHLHLD------------------------------AHGL 376
SmIRX9            C--SKIMV------------WHLHLE------------------------------SQIA 339
Pt006G240200      C--SRVMV------------WLLQLQ------------------------------SSNS 423
Os01g48440        C--TRVMV------------WNFELE------------------------------PPQV 427
Os05g48600        C--NRVMV------------WNFNLE------------------------------PPLL 432
Os10g13810        C--SKIMN------------FHLHLE------------------------------DKNA 325
Os04g01280        C--SQIMN------------WHVPFG------------------------------SESV 362
AtIRX14           CG-RQVLL------------WWLRVE-----------------------------ARADS 432
AtIRX14L          CG-RRVLL------------WWLRVE-----------------------------ARADS 422
Pt007G047500      CG-RQVLL------------WWLRVE-----------------------------ARADS 428
Pt005G141500      CG-RQVLL------------WWLRVE-----------------------------ARADS 423
Os06g47340        CG-KKILL------------WWLRVE-----------------------------ARADS 460
Os04g55670        CG-QAALA------------WSHRSD-----------------------------ALHEV 406
SmIRX14           CG-REVML------------WWIRVE-----------------------------ARADS 459
Hs_GlcAT-I        C--TRVLV------------WHTRTE-------------------------KPKMKQEEQ 321
Hs_GlcAT-P        C--TKILV------------WHTRTE-----------------------------KPVLV 320
Hs_GlcAT-S        C--TKVLV------------WHTRTE------------------------------KVNL 308
                  *     :             :
```
