# Supplementary figures and images for: Site-Directed Mutagenesis of IRX9, IRX9L and IRX14 Proteins Involved in Xylan Biosynthesis: Glycosyltransferase Activity Is Not Required for IRX9 Function in Arabidopsis
Source: PLoS One. 2014 Aug 13;9(8):e105014. doi: 10.1371/journal.pone.0105014 (PMC4132061; doi:10.1371/journal.pone.0105014)

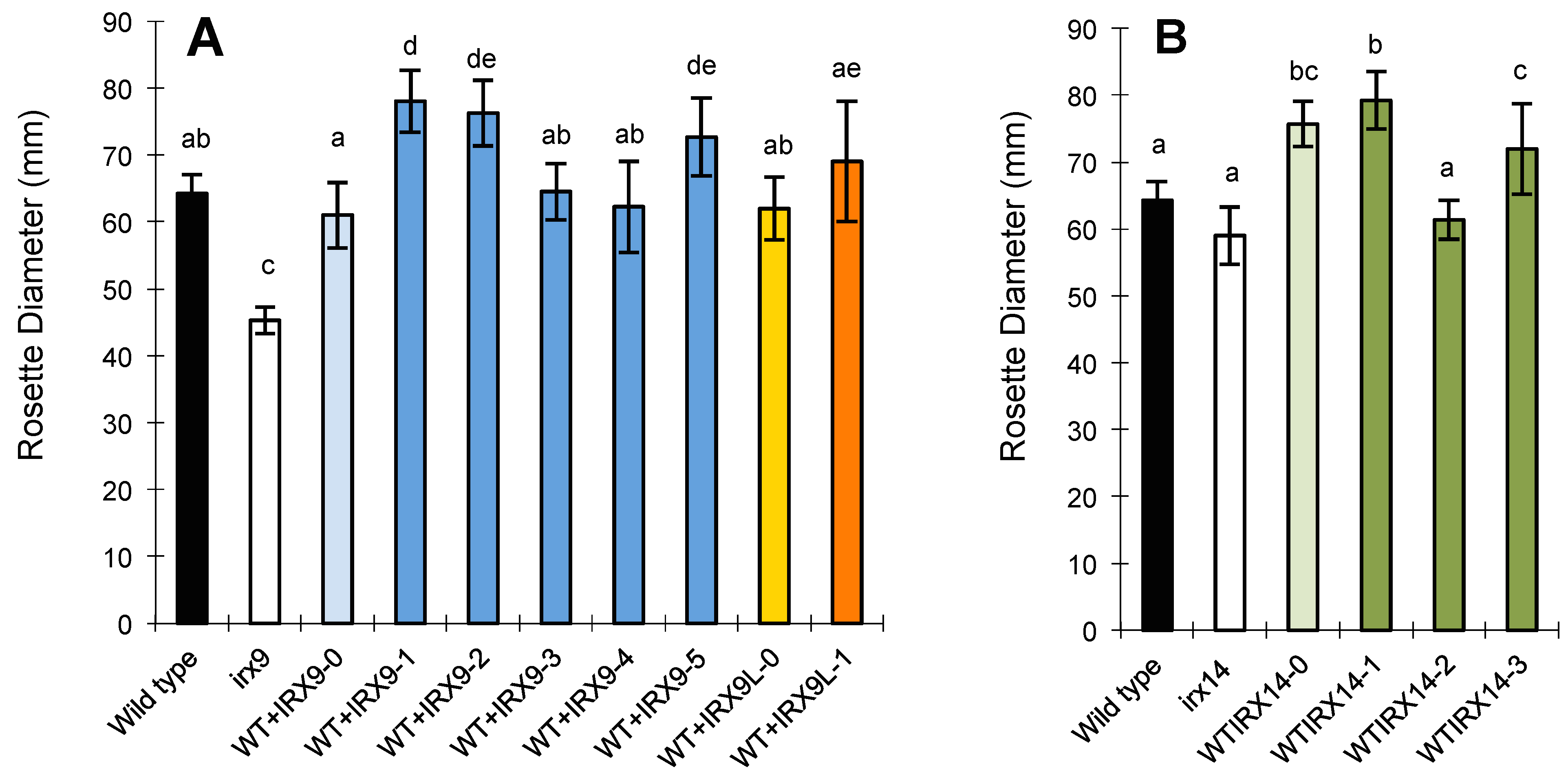

Supplement: Figure S3 — Rosette diameter of 4-week-old wild-type plants transformed with the different constructs. The nomenclature for the constructs used to transform the plants is explained in Table 1. Wild type, irx9, and irx14 were included for comparison. The bars show average ± SD (n = 10). Averages that are not significantly different (ANOVA, Tukey's test, p>0.05) are indicated with the same letter. (TIFF) [file pone.0105014.s003.tiff]

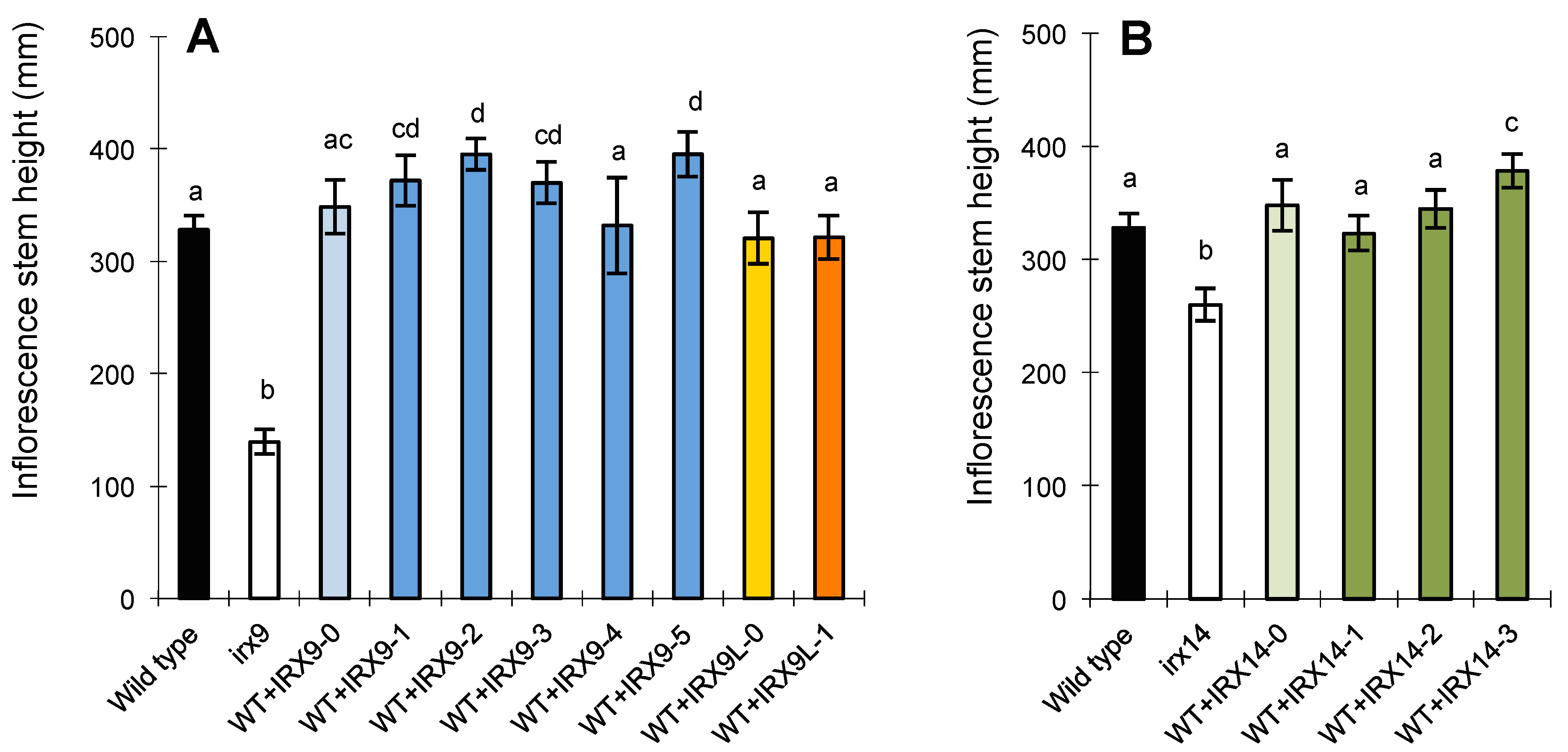

Supplement: Figure S4 — Stem height of 6-week-old wild-type plants transformed with the different constructs. The nomenclature for the constructs used to transform the plants is explained in Table 1. Wild type, irx9, and irx14 were included for comparison. The bars show average ± SD (n = 10). Averages that are not significantly different (ANOVA, Tukey's test, p>0.05) are indicated with the same letter. (TIFF) [file pone.0105014.s004.tiff]

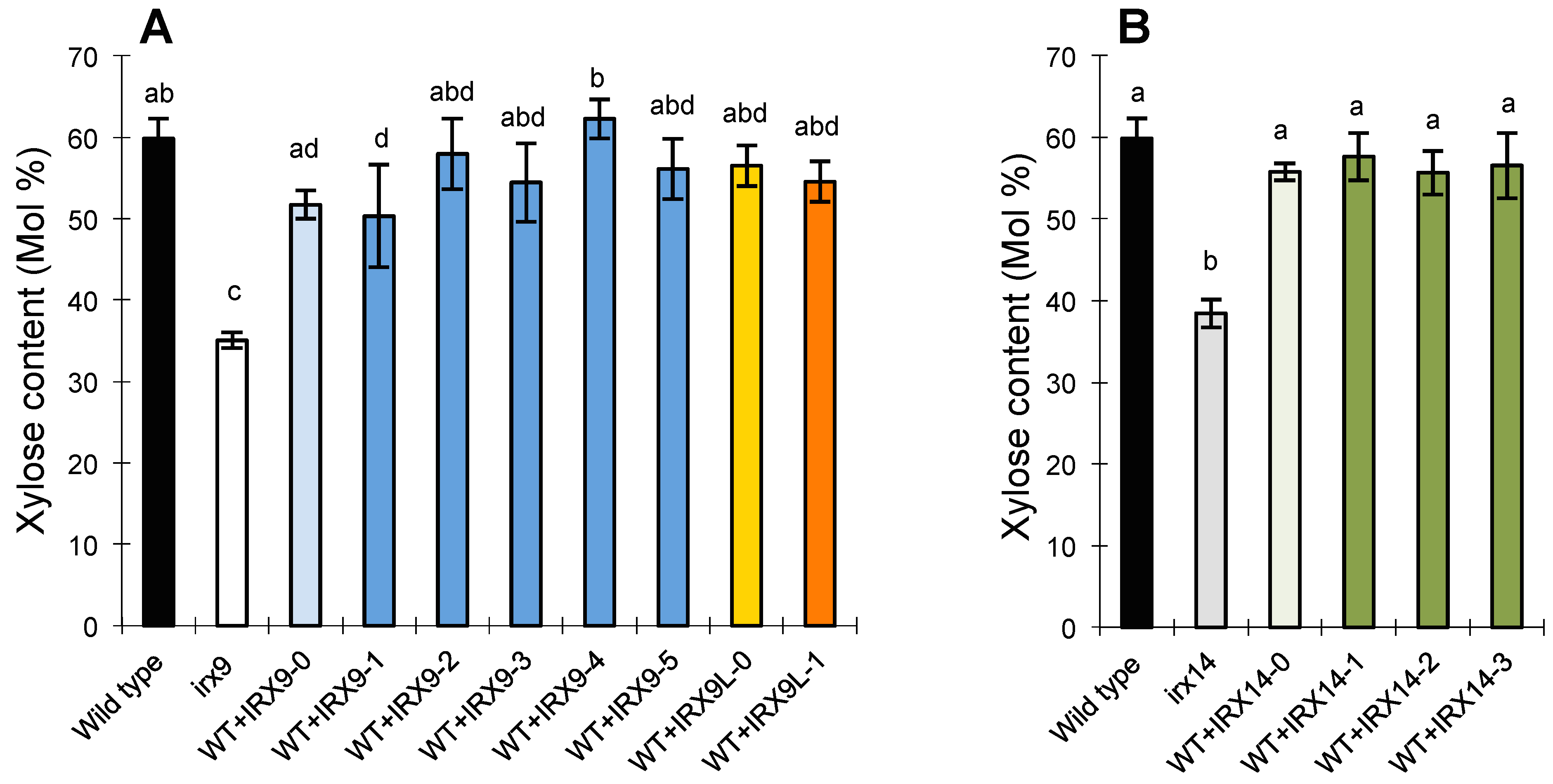

Supplement: Figure S5 — Xylose content in cell wall polysaccharides isolated from stems of 6-week-old wild-type plants transformed with the different constructs. Cell walls were prepared from stems, hydrolyzed in TFA, and the xylose content determined by HPAEC. The nomenclature for the constructs used to transform the plants is explained in Table 1. Wild type, irx9, and irx14 were included for comparison. The bars show average ± SD (n = 4,except for WT+IRX9-4 transformants, where n = 3). Averages that are not significantly different (ANOVA, Tukey's test, p>0.05) are indicated with the same letter. (TIFF) [file pone.0105014.s005.tiff]
