# Supplementary material for: Site-Directed Mutagenesis of IRX9, IRX9L and IRX14 Proteins Involved in Xylan Biosynthesis: Glycosyltransferase Activity Is Not Required for IRX9 Function in Arabidopsis
Source: PLoS One. 2014 Aug 13;9(8):e105014. doi: 10.1371/journal.pone.0105014 (PMC4132061; doi:10.1371/journal.pone.0105014)
Supplement: Table S1 — Primers used for cloning, genotyping and RT-PCR. (PDF) [file pone.0105014.s006.pdf]

Table S1. Primers used for cloning, genotyping and RT-PCR.

**Primers used for generation of point mutations**	
IRX9-1	Fw: 5'-agttccacctcccttattggcgatagtcgtggaaaaacac-3'
	Rv: 5'-gtgtttttccacgactatcgccaataagggaggtggaact-3'
IRX9-2	Fw: 5'-caaattaagcggaatagttcattttgcgtggctaaacaacatatatgatcttgattttt-3'
	Rv: 5'-aaaaatcaagatcatatatgttgtttagccacgcaaaatgaactattccgcttaatttg-3'
IRX9-3	Fw: 5'-catcacaaattaagcggaatagttcattttgcggatgataacaacatatatgatcttgatttttcgtcaaga-3'
	Rv: 5'-tcttgacgaaaaaatcaagatcatatatgttgttatcatccgcaaaatgaactattccgcttaatttgtgatg-3'
IRX9-4	Fw: 5'-ataggaaacgagtggtagtagcagggcctgtttgtg-3'
	Rv: 5'-cacaaacaggccctgctactaccactcgtttcctat-3'
IRX9-5	Fw: 5'-gaagggacttccggcacaagatgcttccaagattatgctttg-3'
	Rv: 5'-caaagcataatcttggaagcatcttgtgccggaagtcccttc-3'
IRX9L-1	Fw: 5'-ggaattgtctactttgctgctgatgccaatatctactcgcttgag-3'
	Rv: 5'-ctcaagcgagtagatattggcatcagcagcaaagtagacaattcc-3'
IRX14-1	Fw: 5'-gatgggattgtgatgtttgcggctgctagtaatatgcatagtatggag-3'
	Rv: 5'-ctccatactatgcatattactagcagccgcaaacatcacaatcccatc-3'
IRX14-2	Fw: 5'-ggagagctcttcgttacctgtagcgggtcctgcttgt-3'
	Rv: 5'-acaagcaggacccgctacaggtaacgaagagctctcc-3'
IRX14-3	Fw: 5'-tggagcctcttggaagcgctggaagacaagttctgc-3'
	Rv: 5'-gcagaacttgtcttccagcgcttccaagaggctcca-3'
**Primers used for genotyping of T-DNA insertions**	
irx9-2	Fw: 5'- gctggtaaggcctcatttttc-3'
	Rv: 5'- aacttaccaacccacccattc-3'
irx14	Fw: 5'-aacgacacgtgtacctccttg-3'
	Rv: 5'- aacatcacaatcccatcaagc-3'
**Primers used to confirm the presence of the transgene**	
IRX9-1/2/3/4/5	Fw: 5'-gcctgtttgtgaatcttcac-3'
	Rv: 5'-accactttgtacaagaaagct-3'
IRX9L-1	Fw: 5'-acaagtttgtacaaaaaagca-3'
	Rv: 5'-accactttgtacaagaaagct-3'
IRX14-1/2/3	Fw: 5'-ggatggataattgatcctc-3'
	Rv: 5'-accactttgtacaagaaagct-3'
**Primers for RT-PCR**	
irx9	Fw: 5'-atgggatctctagagagatca-3'
	Rv: 5'-ggtgcttaaacgtgttcttgt-3'
irx9L	Fw: 5'-cgtcaatccggcgaactc-3'
	Rv: 5'-atttcatagttatgagagcctgcagat-3'
irx14	Fw: 5'-agagttacttaaatcgccggagt-3'
	Fw: 5'-cagtttctttcttgatgcttagacg-3'
ACT2	Fw: 5'-ctcaaagaccagctcttccatc-3'
	Rv: 5'-gcctttgatcttgagagcttag-3'
